# Association between Single Nucleotide Polymorphisms in *SIRT1* and *SIRT2* Loci and Growth in Tibetan Sheep

**DOI:** 10.3390/ani10081362

**Published:** 2020-08-06

**Authors:** Lin-sheng Gui, Sayed Haidar Abbas Raza, Li Zhou, Matthew Garcia, Ayman Hassan Abd El-Aziz, Dawei Wei, Shengzhen Hou, Jianlei Jia, Zhiyou Wang

**Affiliations:** 1 State Key Laboratory of Plateau Ecology and Agriculture, Qinghai University, Xining 810016, Qinghai, China; 2017990039@qhu.edu.cn (L.-s.G.); lgyyuanyi2008@nwafu.edu.cn (L.Z.); 1987990009@qhu.edu.cn (S.H.); 2015990013@qhu.edu.cn (J.J.); 2 National Beef Cattle Improvement Center, Northwest A&F University, Yangling 712100, Shaanxi, China; haiderraza110@nwafu.edu.cn; 3 School of Animal Dairy and Veterinary Sciences, Utah State University, Logan, UT 84322, USA; matthew.garcia@usu.edu; 4 Animal Husbandry and Animal Wealth Development Department, Faculty of Veterinary Medicine, Damanhour University, Damanhour 22511, Egypt; ayman.sadaka@vetmed.dmu.edu.eg; 5 School of Agriculture, Ningxia University, Yinchuan 750021, China; weidawei@nxu.edu.cn

**Keywords:** sirtuins, expression pattern, Tibetan sheep, association analysis, growth-related traits

## Abstract

**Simple Summary:**

In summary, three single nucleotide polymorphisms (SNPs) were observed including two SNPs (g.3148 C > T and g.3570 G > A) in *SIRT1*, and one SNP (g.8074 T > A) in *SIRT2* through sequence analysis. Association analyses suggested that all three SNPs were associated growth-related traits in Tibetan sheep. These findings imply that both *SIRT1* and *SIRT2* may play an important role in growth traits and are potential biomarkers for Marker-assisted selection (MAS).

**Abstract:**

Silent information regulator 1 and 2 (*SIRT1*, 2) were NAD+-dependent histone or non-histone deacetylase, which emerged as key metabolic sensors in several tissues of mammals. In the present study, the search for polymorphisms within the ovine *SIRT1* and *SIRT2* loci as well as association analyses between SNPs and growth-related traits were performed in Tibetan sheep. To determine the expression pattern of *SIRT1* and *SIRT2* genes in Tibetan sheep, the quantitative real-time polymerase chain reaction (qPCR) analysis revealed that those two genes were widely expressed in diverse tissues. Expression of *SIRT1* was less in abomasum of lamb, whereas it was greater in duodenum within adult stage. In the case of *SIRT2*, the greatest expression was observed in reticulum (lamb) and in muscle (adult), whereas the least expression was in liver for lamb and in kidney for adult animals. The association analysis demonstrated that g.3148 C > T polymorphism of *SIRT1* affected heart girth (*p* = 0.002). The g.8074 T > A SNP of *SIRT2* had a significant correlation with body weight (*p* = 0.011) and body length (*p* = 0.008). These findings suggested that the *SIRT1* and *SIRT2* polymorphism was involved in growth-related traits in Tibetan sheep, which may be considered to be genetic markers for improving the growth traits of Tibetan sheep.

## 1. Introduction

The silent information regulators (SIRTs) family, belonging to the nicotinamide adenine dinucleotide (NAD)-dependent deacetylases, was divided into four classes in mammals [1]. Among these, *SIRT1* and *SIRT2* were known as class I in sirtuin family, which exerted diverse influence on lifespan, insulin resistance, and metabolism [2].

Mammalian *SIRT1* localized in the nucleus was in agreement with its effect on histone deacetylase [3]. In the arcuate nucleus of mouse, overexpressing *SIRT1* stimulated energy expenditure by improving leptin sensitivity in adipose tissue, and suppressed food intake, which prevented age-associated weight gain [4]. In response to high-fat diet, accelerated adiposity, exacerbated insulin resistance, and severe brown adipose tissue degeneration phenotype were displayed in *SIRT1*+/−mice. This was likely to occur via down-regulation of thermogenic genes expression (i.e., UCP 1 and PPAR γ) [5]. Compared with *SIRT1, SIRT2* was localized mainly in the cytoplasm [6]. Inhibition of endogenous *SIRT2* resulted in amelioration of insulin sensitivity in C2C12 cells [7]. By regulation of phosphoenolpyruvate carboxykinase 1 (GTP 1) and glutaminase (PAG), *SIRT2* was involved in mitochondrial metabolism, leading to impairment of energy metabolism in hepatocellular carcinoma cells [8]. Knockdown of *SIRT2* contributed to liver insulin resistance in mice, and increased food intake in response to high-fat diet [9]. Besides, *SIRT2*−/−mice showed mitochondrial depletion, which caused redox dyshomeostasis and energy failure, suggesting *SIRT2* acted as a mediator of metabolic regulation [10]. Both *SIRT1* and *SIRT2* focused on their function of regulated energy homeostasis [11]. However, there were no reports on associations between those genes and growth traits in Tibetan sheep. This paper aimed to explore the role of *SIRT1* and *SIRT2* in Tibetan sheep by analyzing association between single nucleotide polymorphisms (SNPs) and growth traits.

## 2. Materials and Methods

Thirteen tissues of Tibetan sheep were collected from three adult individuals of 1-year-old and three 7-days-old lambs, respectively. All samples were purchased from the Institute of Animal Husbandry and Veterinary Medicine, Haibei state, Qinghai province, China. All animal procedures for experiments were approved by Committee of Experimental Animal Management (EAMC) at Qinghai University, in accordance with the code EAMC/20-556. Moreover, the use of experimental animals was carried out in accordance to the rules and guidelines of the organization and government.

Total RNA was extracted, and reverse transcribed by a Total RNA kit and PrimeScriptTM RT Reagent Kit (TaKaRa, Dalian, China), respectively. The relative expression level of *SIRT1* (NC_040276.1) and *SIRT2* (NC_040265.1) was calculated using Applied Biosystems 7500 Fast Real-Time PCR System (Applied Biosystems, Foster City, CA, USA) with the 2^−△△Ct^ method. The results of qPCR were normalized by the expression of β-actin (NM_001009784.3) and GAPDH (NM_001190390.1). Relative mRNA expression level was presented as mean ± SD (n = 3).

The investigated ewe with a total of 402 ewes (12 months age, averagely) collected from the demonstration area of ecological animal husbandry (Haiyan county, Qinghai Province, China). Individuals were raised on a diet of corn and corn silage, according to nutrient requirements of growing sheep (NRC, 2012). All ewes were offered oat hay ad libitum in management process under the same ratio of roughage to concentrate (7:3) and similar rearing environment, such as similar temperature, altitude, humidity, etc.

Synchronously, body weight, withers height, heart girth, and body length were recorded. The Omgam Blood DNA Kit (Omgam Bio-Tek, Doraville, GA, USA) was utilized to isolate genomic DNA from whole blood samples. On the basis of the sequence of the ovine *SIRT1* (NC_040276.1) and *SIRT2* (NC_040265.1), PCR primers were designed via Primer Premier Software (Version 4.0). Detailed information of primers was depicted in Table 1. PCR amplification was performed according to the procedure of Sun et al. [12], and then sequenced using an ABI 3730 sequencer (ABI, Foster City, CA, USA).

Gene frequencies, Hardy-Weinberg equilibrium (HWE), and polymorphism information content (PIC) were computed using POPGENE software (Version 3.0). Linkage disequilibrium (LD) and haplotype construction were calculated using the web-based tool (http://analysis.bio-x.cn/).

Statistical analysis was conducted by SPSS 20.0 (IBM Company, New York, NY, USA). The general linear model (GLM) was used to analyze the association between SNPs and growth traits in Tibetan sheep. The basic linear model was: Yij = µ + Gi + Aj + eij, Gi was the fixed effect of genotype, Aj was the fixed effect of age, and eij was the random error.

For a more detailed review of the results, we corrected the *p* values by Bonferroni correction, which uses a modified criterion for significance (a/k, where a = 0.05, and k is the overall number of independent statistical tests conducted on the given data). For *SIRT1*, we analyzed four traits and two different SNPs, resulting in an adjusted *p*-value of 0.00625 for the 5% significance threshold. For *SIRT2*, we analyzed four traits and one SNP, resulting in an adjusted *p*-value of 0.0125 for the 5% significance threshold.

## 3. Results

### 3.1. Expression Levels of SIRT1 and SIRT2 in Tissues

**Figure 1:**
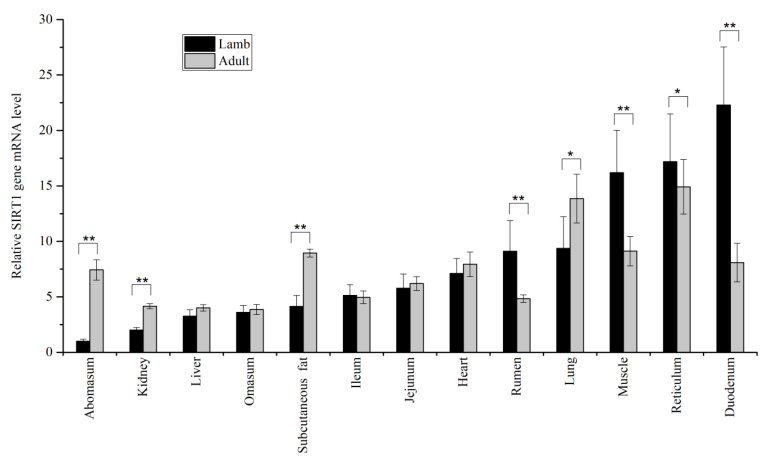
The relative mRNA expression of ovine *SIRT1*. * *p* < 0.05, ** *p* < 0.01.

**Figure 2:**
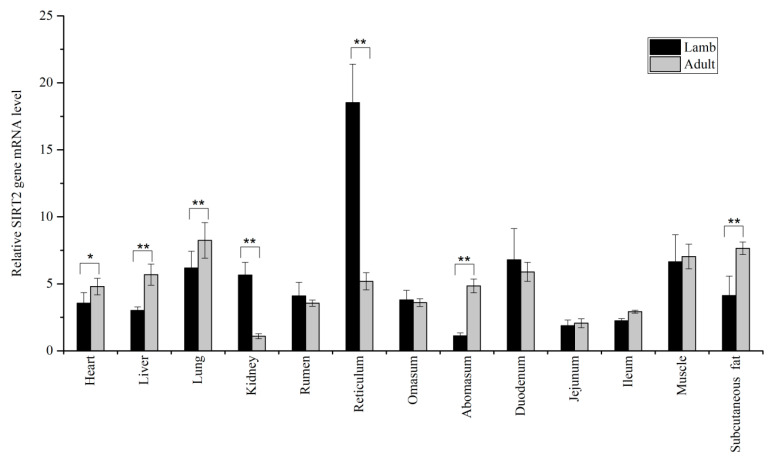
The relative mRNA expression of ovine *SIRT2*. * *p* < 0.05, ** *p* < 0.01.

The result showed the mRNA level of *SIRT1* and *SIRT2* in thirteen different tissues with within two different age stages. The results presented in Figure 1 demonstrated ovine *SIRT1* was primarily expressed in diverse tissues. Remarkably higher mRNA expression was detected in the abomasum (*p* < 0.01), kidney (*p* < 0.01), subcutaneous fat (*p* < 0.01), and lung (*p* < 0.05) as compared with adult ewes, whereas the opposite result was found in the rumen (*p* < 0.01), muscle (*p* < 0.01), reticulum (*p* < 0.05), and duodenum (*p* < 0.01). As showed in Figure 2, when comparing expression of *SIRT2* of adult ewes against lamb, the expression of *SIRT2* in kidney and reticulum was greater in lambs than in adult ewes (*p* < 0.01). In the heart (*p* < 0.05), liver (*p* < 0.05), lung (*p* < 0.01), subcutaneous fat (*p* < 0.01), and abomasum (*p* < 0.01), it was greater in adult ewes than in lambs.

### 3.2. Polymorphisms and Genetic Diversity

The PCR amplified fragments were directly sequenced. A total of three SNPs were revealed, including two polymorphic loci (g.3148C > T and g.3570 G > A) within *SIRT1* intron 2 and 3, respectively, and one polymorphic locus (g.8074 T > A) was detected in the exon 7 of *SIRT2*.

As displayed in Table 2, the predominant frequencies of alleles were C of g.3148 C > T, G of g.3570 G > A, and T of g.8074 T > A. According to the results of χ^2^ test, the genotypes of three SNPs were consistent with HWE (*p* > 0.05). In addition, the PIC values of g.3148 C > T and g.3570 G > A were 0.30 and 0.26, respectively, indicating medium genetic diversity in *SIRT1* loci. Whereas, g.8074 T > A locus in *SIRT2* exhibited low genetic diversity.

### 3.3. LD Analysis

To analyze the linkage relationships between the g.3148 C > T and g.3570 G > A, LD value was estimated. The *r*^2^ value was 0.005 (<0.33), indicating weak linkage between the two loci in *SIRT1*. As was presented in Table 3, four estimated haplotypes were detected as the prominent haplotypes in the Tibetan sheep populations. The frequency of Hap2 haplotype (-CG-) was 62.20%, followed by Hap4 haplotype (-TG-), Hap1 haplotype (-CA-), and Hap3 haplotype (-TA-), with 18.6, 13.30, and 5.90%, respectively.

### 3.4. Association Analysis

Table 4 illustrated the association results between the identified SNPs in *SIRT1/2* and growth traits. Compared with animals with genotype TT, the role of CC and CT genotype at g.3148 C > T locus caused the highest average for heart girth (*p* = 0.002). At g.8074 T > A locus, individuals with the AA genotype tended to have larger body weight (*p* = 0.011) and body length (*p* = 0.008) than individuals with the TA and TT genotype. Multiple effects of the 2 SNPs of *SIRT1* were evaluated, and a total of nine haplotype combinations were identified (data not shown). Combinations with frequencies lower than 5.0% were neglected and the remaining combinations were selected for further analysis. As presented in Table 5, when the combination results were compared, no significant differences were detected between the combined haplotype of these two SNPs of *SIRT1* and growth traits in Tibetan sheep (*p* > 0.003).

## 4. Discussion 

Tibetan sheep (*Ovis aries*), providing hides, meat, and milk for indigenous Tibetans [13], possessed hypoxia-tolerant and cold-resistant ability in harsh Tibetan plateau [14]. According to the biological function in energy metabolism, ovine *SIRT1* and *SIRT2* may exert a critical influence on regulation of growth traits in Tibetan sheep.

The qPCR results revealed that *SIRT1* was primarily expressed, consistent with the researches of other species [15,16]. Specifically, the predominant expression of *SIRT1* gene existed in abomasum, kidney, subcutaneous fat, and lung at adult stage, whereas it was greater in rumen, muscle, reticulum, and duodenum of lambs. The expression pattern of ovine *SIRT1* significantly differed with what was reported from cattle [17]. This was because that bovine *SIRT1* was more predominant in muscular tissue than in adipose tissue [18]. The difference in expression may be a result of the metabolic diversity among species. Similarly, *SIRT2* was extensively expressed in different tissues in Tibetan sheep. Previous observations indicated that gene expression levels might, at least in part, parallel well with its corresponding function in mammal [19]. When comparing expression of *SIRT2* within two diverse age stages, the *SIRT2* expression level in liver, lung, abomasum, and subcutaneous fat were significantly increased with advancing age, implying *SIRT2* exhibited crucial molecular function of adult more than lamb in those tissues.

In this study, the g.3148 C > T and g.3570 G > A were identified in the intron of *SIRT1*, which contributed to the genetic breeding of Tibetan sheep. Although the structure of the encoded protein was never changed, intronic SNPs maybe effected on metabolism of mRNA or assembly of spliceosome components [20], thereby reducing genetic mutation and maintaining the genetic stability. Several introns contained enhancers or *cis*-acting elements that promoted the initiation and extension of transcription, thus affecting gene expression levels [21]. Additionally, introns promoted the frequency of recombination between genes via increasing length of gene sequence [22]. In the current study, it is tempting to speculate that intronic SNPs with ovine *SIRT1* may impact the biological function, resulting in an alteration in phenotype of mammal.

Previously, the g.2694 C > T and g.3801 T > C mapping on intronic SNPs of max dimerization protein 3 (MXD3) influenced on several growth traits in two Chinese indigenous beef cattle [23]. The intronic g.18341 C > T of the lipoprotein lipase (LPL) was significantly correlated with withers height and chest depth in Nanyang cattle [24].

Accumulating observations indicated that the *SIRT2* was an excellent candidate gene involved in economic characters of ruminant. In Chinese Nanyang cattle, the g.17333 C > T and g.17578 A > G in *SIRT2* significantly affected 18-months-old body weight [25]. The g.19676 G > A in the 3’UTR of *SIRT2* was associated with an alteration in growth traits of Qinchuan cattle [26]. Additionally, a novel 7-bp indel of *SIRT2* was corrected with the body length in Chinese Jiaxian cattle [27]. In the present study, a SNP (g.8074 T > A) of *SIRT2* significantly influenced the growth-related traits in Tibetan sheep, which corresponded with the function in energy metabolism of *SIRT2*.

## 5. Conclusions 

In summary, three SNPs were observed including two SNPs (g.3148 C > T and g.3570 G > A) in *SIRT1*, and one SNP (g.8074 T > A) in *SIRT2* using DNA direct sequencing. Both g.3148 C > T and g.8074 T > A were associated growth-related traits in Tibetan sheep. These findings demonstrated that *SIRT1* and *SIRT2* might exert a crucial influence on growth traits. Further research should be conducted in a large population and different population before applying those genea to molecular marker-assisted selection.

## Figures and Tables

**Table 1:** Primers used in these experiments.

Name	Primer Sequence (5’ to 3’)	Tm (°C)	Product Location	Product Length
*SIRT1*-SNP	CCTTAGCTCTGAAGATGTTCT	55.7	Part of intron 2, exon 3, and part of intron 3	629 bp
	TACTGTGTCAATAAATAGAA			
*SIRT2*-SNP	TTTTGGAGGAATGTTTGATG	57.0	Part of exon 5, intron 5, exon 6, intron 6, exon 7, and part of intron 7	764 bp
	ACTATGCTTCAAGGCTTGCA			
*SIRT1*-qPCR	ACTTCTACGACGACGACGACGAG	61.0	-	182 bp
	CCGAGGTCTTGGAGTCCAGTCAC			
*SIRT2*-qPCR	TGGCGGAGAAGCAGAGATGGAC	61.0	-	116 bp
	TGTAGCGGCTCACTCCTTCCAG			
*β*-actin-qPCR	CGTCCGTGACATCAAGGAGAAGC	61.0	-	143 bp
	GGAACCGCTCATTGCCGATGG			
GAPD-qPCR	CGGCACAGTCAAGGCAGAGAAC	61.0	-	115 bp
	CACGTACTCAGCACCAGCATCAC			

**Table 2:** Population genetic analysis of SNPs in *SIRT1* and *SIRT2*.

Loci (Gene)	Genotypic Frequency (%)			PIC	Maximum Allele Frequency (%)	HWE
g.3148 C > T (*SIRT1*)	CC	CT	TT			
	58.46	34.08	7.46	0.30	75.50 (C)	*p* > 0.05
g.3570 G > A (*SIRT1*)	GG	GA	AA			
	67.16	27.36	5.48	0.26	80.85 (G)	*p* > 0.05
g.8074 T > A (*SIRT2*)	TT	TA	AA			
	72.39	23.88	3.73	0.23	84.33 (T)	*p* > 0.05

Polymorphism information content, PIC. Hardy-Weinberg equilibrium, HWE.

**Table 3:** Frequencies analysis of *SIRT1* haplotypes.

Haplotype	g.3148 C > T	g.3570 G > A	Frequency
Hap1	C	A	13.30%
Hap2	C	G	62.20%
Hap3	T	A	5.90%
Hap4	T	G	18.60%

**Table 4:** Association of genotypes of SNPs in *SIRT1* and *SIRT2* with growth traits in Tibetan sheep.

Loci (Gene)	Genotypes (N)	Body Weight (kg)	Withers Height (cm)	Body Length (cm)	Heart Girth (cm)
g.3148 C > T (*SIRT1*)	CC (235)	58.63 ± 0.46	71.60 ± 0.82	75.00 ± 0.65	96.08 ± 1.03 ^b^
	CT (137)	56.24 ± 0.59	70.54 ± 0.57	73.83 ± 0.54	96.73 ± 0.79 ^b^
	TT (30)	54.48 ± 0.79	70.78 ± 0.68	72.51 ± 0.77	88.38 ± 0.85 ^a^
*p^1^*		0.072	0.253	0.109	0.002
g.3570 G > A (*SIRT1*)	GG (270)	58.54 ± 0.65	71.62 ± 0.76	75.03 ± 0.82	96.77 ± 0.88
	GA (110)	55.88 ± 0.48	70.32 ± 0.59	73.39 ± 0.69	94.15 ± 0.72
	AA (22)	52.94 ± 0.72	70.01 ± 0.60	72.02 ± 0.66	90.78 ± 0.99
*p^1^*		0.038	0.157	0.070	0.019
g.8074 T>A (*SIRT2*)	TT (291)	57.53 ± 0.27 ^b^	71.17 ± 0.20	74.44 ± 0.19 ^b^	96.03 ± 0.29
	TA (96)	56.09 ± 0.47 ^b^	70.41 ± 0.35	73.23 ± 0.33 ^b^	94.43 ± 0.53
	AA (15)	66.07 ± 0.92 ^a^	76.22 ± 0.89	81.63 ± 0.83 ^a^	98.00 ± 1.17
*p^2^*		0.011	0.031	0.008	0.074

Values were showed as the least squares means ± standard error. ^a,b^ Means with different superscripts are significantly different (*p^1^* < 0.00625 and *p^2^* < 0.0125) after Bonferroni correction.

**Table 5:** Association of different genotypes of SNPs in *SIRT1* with growth traits in Tibetan sheep.

Genotypes (N)	Body Weight (kg)	Withers Height (cm)	Body Length (cm)	Heart Girth (cm)
Hap2/1 (63)	56.84 ± 0.44	70.59 ± 0.63	74.03 ± 0.45	95.52 ± 0.73
Hap2/2 (159)	59.72 ± 0.67	72.08 ± 0.70	75.59 ± 0.43	96.63 ± 1.23
Hap2/3 (34)	55.61 ± 0.48	70.12 ± 0.79	73.17 ± 0.57	94.60 ± 0.88
Hap2/4 (99)	56.50 ± 0.40	70.64 ± 0.53	74.03 ± 0.61	97.67 ± 0.74
*p^1^*	0.032	0.225	0.438	0.040

Values were showed as the least squares means ± standard error.
